# Feasibility of Intensity-Modulated and Image-Guided Radiotherapy for Functional Organ Preservation in Locally Advanced Laryngeal Cancer

**DOI:** 10.1371/journal.pone.0042729

**Published:** 2012-08-20

**Authors:** Nam P. Nguyen, Alexander Chi, Michael Betz, Fabio Almeida, Paul Vos, Rick Davis, Benjamin Slane, Misty Ceizyk, Dave Abraham, Lexie Smith-Raymond, Michelle Stevie, Siyoung Jang, Steven Gelumbauskas, Vincent Vinh-Hung

**Affiliations:** 1 Department of Radiation Oncology, University of Arizona, Tucson, Arizona, United States of America; 2 Department of Radiation Oncology, University of West Virginia, Morgantown, West Virginia, United States of America; 3 Department of Radiation Oncology, University Hospitals of Geneva, Geneva, Switzerland; 4 Southwest PET/CT Institute, Tucson, Arizona, United States of America; 5 Department of Biostatistics, East Carolina University, Greenville, North Carolina, United States of America; 6 Department of Radiation Oncology, University of Pittsburgh, Pittsburgh, Pennsylvania, United States of America; Johns Hopkins University, United States of America

## Abstract

**Purpose:**

The study aims to assess the feasibility of intensity-modulated and image-guided radiotherapy (IMRT, and IGRT, respectively) for functional preservation in locally advanced laryngeal cancer. A retrospective review of 27 patients undergoing concurrent chemoradiation for locally advanced laryngeal cancers (8 IMRT, 19 IGRT) was undertaken. In addition to regular clinical examinations, all patients had PET imaging at 4 months and 10 months after radiotherapy, then yearly. Loco-regional control, speech quality and feeding-tube dependency were assessed during follow-up visits.

**Results:**

At a median follow-up of 20 months (range 6–57 months), four out of 27 patients (14.8%) developed local recurrence and underwent salvage total laryngectomy. One patient developed distant metastases following salvage surgery. Among the 23 patients who conserved their larynx with no sign of recurrence at last follow-up, 22 (95%) reported normal or near normal voice quality, allowing them to communicate adequately. Four patients (14.8%) had long-term tube feeding-dependency because of severe dysphagia (2 patients) and chronic aspiration (2 patients, with ensuing death from aspiration pneumonia in one patient).

**Conclusions and Clinical Relevance:**

Functional laryngeal preservation is feasible with IMRT and IGRT for locally advanced laryngeal cancer. However, dysphagia and aspiration remain serious complications, due most likely to high radiation dose delivery to the pharyngeal musculatures.

## Introduction

Locally advanced laryngeal cancer was traditionally treated with total laryngectomy followed by postoperative radiotherapy given the high risk of loco-regional recurrences [1]. However, the presence of a stoma following total laryngectomy is often associated with chronic airway irritation and coughing, sleep difficulties and poor cosmesis [2]–[4]. Additionally, resection of large tumors and neck dissection may result in substantial chronic postoperative pain [5], [6]. In an attempt to improve patient quality of life, the Veterans Administration Laryngeal Study (VALG) first investigated the feasibility of an organ-preservation approach, achieving a 64% rate of larynx preservation with induction chemotherapy followed in responders by definitive radiotherapy, while maintaining a survival rate comparable to that seen in laryngectomized patients [7]. The Radiation Therapy Oncology Group (RTOG) 91–11 Study subsequently demonstrated an even higher rate of laryngeal preservation with concurrent chemoradiation rather than induction chemotherapy followed by radiotherapy alone at least for patients with T3 disease [8]. While the exclusion of patients with T4 lesions from RTOG 91-11 left it unclear whether laryngeal preservation with concurrent chemoradiation was feasible for all patients with locally advanced laryngeal cancer for functional laryngeal preservation, later studies confirmed the feasibility of such an approach for T4 disease as well [9], [10]. Nevertherless, persistent concerns remain about the functional consequences of delivering curative radiation doses to the larynx and pharyngeal musculature, with the risk of ensuing chronic edema and fibrosis, leading to voice alteration and chronic dysphagia and potentially defeating the purpose of laryngeal preservation [11], [12]. The traditional treatment for locally advanced laryngeal cancer has been two lateral fields matched with an anterior supraclavicular field. This three-dimensional (3-D) radiotherapy technique did not allow sparing of normal tissues which received the same tumor dose. Xerostosmia secondary to radiation related destruction of the parotid glands, osteoradionecrosis secondary to high mandibular dose, and hearing loss from cochlea damage, are common sequellaes of 3-D conformal radiotherapy (3-DRT) leading to poor patient quality of life [13]–[15]. Recently, intensity-modulated (IMRT) and image-guided radiotherapy (IGRT) have been introduced in the treatment of head and neck cancer to decrease treatment toxicity by improving normal tissue sparing [16]–[18]. Rapid dose fall off away from the target allows significant sparing of the normal organs at risk for complications and potentially improves patient quality of life with these new radiotherapy techniques. The simultaneously integrated boost (SIB) technique of IMRT and IGRT also allows selective dose assignment to different target levels with the tumor receiving the highest dose and subclinical disease treated to a lower dose, thus allows more control of the radiation dose received by the normal organs adjacent to the target. Preliminary dosimetric studies of laryngeal cancer also demonstrated improved dose conformality of IMRT compared to 3-DRT, potentially leading to improved tumor control if the tumor dose can also be increased with the integrated boost technique [19]–[20]. However, the advantages of IMRT and IGRT may also be negated because of possible marginal miss if the tumor volume is not clearly defined [21]. Thus, accurate pre-treatment imaging such as positron emission tomography (PET) scans is almost mandatory for laryngeal cancer because of its superior tumor delineation compared to CT scans [22]. Following radiotherapy, high dose to the larynx for tumor control often lead to severe laryngeal edema which may be interpreted as tumor recurrence. The ability of PET scans and PET-CT to distinguish tumor recurrence from laryngeal edema and fibrosis also make it an ideal tool for patient follow-up to avoid unnecessary laryngeal biopsies which may cause chondronecrosis [23]–[24]. We investigate in this retrospective study the feasibility and potential benefit of IMRT and IGRT for anatomic and functional organ preservation in combination when FDG-PET has been used to guide target volume felineation, and post treatment surveillance. Table 1 illustrates the potential benefits and possible pitfalls of IMRT and IGRT compared to 3-D conformal radiotherapy.

**Table 1:** Potential advantages and pitfalls of intensity-modulated radiotherapy (IMRT) and image-guided radiotherapy (IGRT) compared to 3-dimentional conformal radiotherapy (3-D CRT).

	IMRT, IGRT	3-D CRT
Advantages:	Sparing of normal organs because of rapid dose fall	No sparing
	Tumor dose escalation possible because of different dose levels within the target volume	No dose escalation
	Visualization of tumor shrinkage feasible for IGRT allowing re-planning during radiotherapy	No visualization
Pitfalls:	Marginal miss if tumor not properly contoured leading to local recurrences.	Unlikely

## Materials and Methods

The medical records of 27 patients undergoing curative radiotherapy for locally advanced laryngeal cancers at the University of Arizona Radiation Oncology Department were retrospectively reviewed following institutional review board (IRB) approval. The University of Arizona IRB waived the patient consent requirement because of the retrospective nature of the study limited to chart reviews. The patient information was de-identified to protect patient confidentiality. Eight patients were treated with the whole field (WF) IMRT technique on the Elekta linear accelerator (6 MV photons) with 7 to 9 radiation beams from February 2007 to December 2008. Following installation of the helical Tomotherapy unit in December 2008, 19 patients were treated with the WF IGRT technique. Prior to treatment, each patient was simulated in the supine position with a head and neck aquaplast mask for treatment immobilization. A computed tomography (CT) scan with and without intravenous (IV) contrast for treatment planning was performed in the treatment position. The head and neck areas from the vertex to the mid thorax were scanned with a slice thickness of 3 mm CT scan with IV contrast was employed to outline the tumor and grossly enlarged cervical lymph node for target volume delineation. Radiotherapy planning was performed on the CT scan without contrast to avoid possible interference of contrast density on radiotherapy isodose distributions. Diagnostic positron emission tomography (PET)-CT scan planning for tumor imaging was also incorporated with CT planning. A 0.5 cm bolus material was placed on any area of the skin involved by the tumor and on any palpable cervical lymph nodes. Patients treated on Tomotherapy also had 0.5 cm bolus over the thyroid cartilage to prevent possible underdosing of the larynx. Normal organs at risk for complication were outlined for treatment planning (spinal cord, brain stem, bilateral cochlea, mandible, parotid glands, bilateral eyes, and oral cavity). Radiation therapy dose was similar for patients in both groups using the integrated boost technique to decrease treatment toxicity. The tumor and grossly enlarged lymph nodes (CTV1) on CT scan with a margin (PTV1) were treated to 70 Gy in 35 fractions (2 Gy/fraction). The margins were 5 mm to 1 cm all around CTV1 depending on anatomic location. The areas at high risk-PTV2 (at least 1 cm around gross tumor and pathologic cervical lymph nodes) and low risk -PTV3 (subclinical regional lymph nodes with 5 mm margins) for tumor spread were treated respectively to 63 Gy and 56 Gy in 35 fractions, respectively. Minimal target coverage was 95% of the prescribed dose for all targets with at least 99% of the prescribed dose delivered to gross tumor and involved cervical lymph nodes. The lymph nodes in the ipsilateral neck including the retropharyngeal lymph nodes were treated to the base of skull if there was any cervical lymph node enlargement (or PET-positive lymph nodes). Contralateral uninvolved lymph nodes were treated prophylactically with the C1 vertebrae as the superior border. In cases of bilateral cervical lymph node involvement, the bilateral neck was treated to the base of skull to avoid any marginal miss. Mean dose to the parotid was kept below 2600 cGy if there was no ipsilateral cervical lymph node enlargement. Dose constraints for other normal organs at risk (OAR) for complications were: spinal cord (45 Gy), brain stem (50 Gy), optic chiasm (45 Gy), mandible (70 Gy to less than 30% of the mandible).

Concurrent chemoradiation was recommended for all patients. The type of chemotherapy regimen was left at the discretion of the medical oncologist depending on patient functional status and co-morbidities. Prophylactic percutaneous gastrostomy (PEG) feeding-tube placement was also recommended for all patients prior to treatment given expected weight loss secondary to acute toxicity including mucositis and dysgueusia. Weekly complete blood count (CBC) and blood chemistry work-ups were performed during chemoradiation. Treatment breaks and weight loss were recorded during chemoradiation. Acute and long-term toxicities were graded according to Radiotherapy Oncology Group (RTOG) group criteria severity scale (http://ctep.cancer.gov).

After treatment, all patients had follow-up visits at one month, then, every three months thereafter. Clinical and direct laryngoscopic examination were performed at each follow up visit. PET scan, or PET-CT imaging was performed at four months and ten months, then yearly after treatment. All PET-positive areas were biopsied to detect recurrence and total salvage laryngectomy (TL) with bilateral neck dissection (BND) carried out, if biopsy was positive. Voice quality (ability to conduct a normal conversation over the phone or ability to communicate without difficulty at work) was assessed at each follow-up visit. Patient ability to resume normal oral feeding and feeding-tube dependency was also evaluated at each visit.

Survival analysis was analyzed using Kaplan-Meier estimation.

## Results

**Figure 1:**
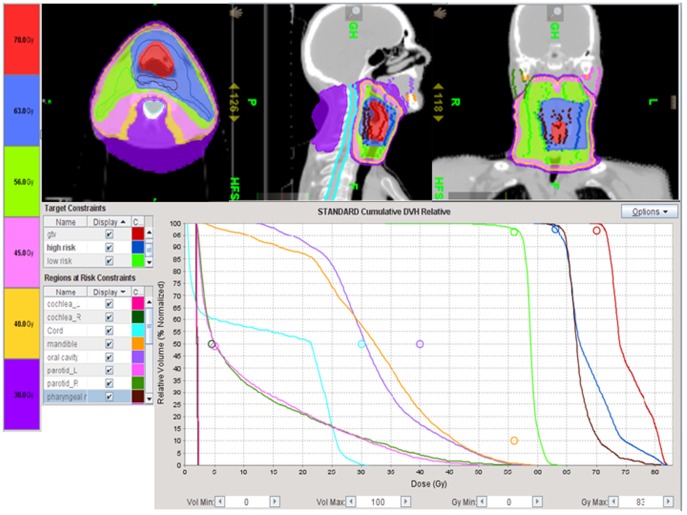
Dose-volume .histogram in a patient with T3N0M0 supraglottic laryngeal cancer illustrating the potential of image-guided radiotherapy to deliver a high radiation dose to the gross tumor while sparing the radiosensitive normal organs. Mean tumor dose: 75.4 Gy; mean right parotid dose (light green):11.4 Gy; mean left parotid dose (violet): 11.3 Gy; mean right (dark green) and left (pink) cochlea dose: 2 Gy; maximum mandibular dose (orange): 59.5 Gy; maximum spinal cord dose (light blue): 31.2 Gy. The radiation dose to these normal structures was well below the threshold for normal tissue damage and could potentially improve the patient quality of life after treatment. The red color illustrated radiation dose (70 Gy) to the gross tumor volume (gtv) while the dark blue and green color demonstrated radiation dose to the high risk area (63 Gy) and low risk area (56 Gy) respectively. The brown color illustrated radiation dose to the pharyngeal muscles which was high (mean 67 Gy) because of the close proximity to the gross tumor and may explain the high rate of dysphagia following radiotherapy for locally advanced laryngeal cancer.

We identified 27 patients with loco-regionally advanced invasive squamous cell carcinoma of the larynx treated at the University of Arizona Radiation Oncology department from 2007 to 2010. Median age at diagnosis was 63 years-old (range: 52–84 years-old). There were 25 males and 2 females. Seventeen patients had stage III, nine had stage IVA, and one had stage IVB disease. Twenty six patients received concurrent chemoradiation, while one patient who declined concurrent chemotherapy received radiotherapy alone. Table 2 summarizes patient characteristics. Table 3 summarizes radiation dose to the larynx and other head and neck organs. Figure 1 illustrates the dose-volume histogram of a patient with a T3N0M0 supraglottic laryngeal cancer treated with IGRT demonstating the feasibility of this new technique of radiotherapy to spare the normal head and neck organs from excessive radiation.

**Table 2:** Patient characteristics.

	IGRT	IMRT	Total
Patient No	19	8	27
Age			
Median	60	68	63
Range	52–78	60–84	52–84
Sex			
Male	17	8	25
Female	2	0	2
Squamous histology	19	8	27
Sites			
Glottis	5	6	11
Supraglottis	14	2	16
Stages			
III	11	6	17
IVA	7	2	9
IVB	1	0	1
T stages			
T1	0	2	2
T2	5	2	7
T3	9	3	12
T4	5	1	6
Neck nodes			
N0	12	4	16
N1	4	3	7
N2	3	1	4
Treatment			
Radiotherapy alone	1	0	1
Chemoradiation	18	8	26

IGRT: Image-guided radiotherapy; IMRT: Intensity-modulated radiotherapy.

**Table 3:** Dose distribution in Gray (Gy) to the larynx and other head and neck organs.

	Mean (range)	Max (range)
Larynx	72.5 (70.5–76.7)	76.1 (71.9–82.2)
Spinal cord		39.1 (28.9–44.6)
Mandible	37.9 (23–56.1)	64 (46.4–74.8)
Right TMJ	12.8 (1.6–41)	34.9 (2.1–46.6)
Left TMJ	9.7 (1.8–23)	22.4 (2.3–44.7)
Right parotid	27.7 (10–40.7)	
Left parotid	32.4 (14.4–40.5)	
Right cochlea	9.4 (1–35)	11.9 (1.9–44.9)
Left cochlea	8.9 (1–35.9)	17.5 (1–47.1)

TMJ: Temporo-mandibular joint.

Chemotherapy consisted of cisplatin (P) 30 mg/m2 intravenously (IV) weekly in 12 patients and cisplatin 100 mg IV on day 1, 22, and 43 of radiotherapy in 11 patient. Four patients had induction chemotherapy with taxotere (T) 75 mg/m2 IV, cisplatin 100 mg/m2 IV followed by 5-fluorouracil (F) 1000 mg/m2 for four days, repeated every three weeks for two cycles, followed during radiotherapy by carboplatin IV weekly with an area under the curve of 1.5.

At a median follow-up of 20 months (6–57 months), four patients developed local recurrence and underwent salvage TL and BND. One of the four patients was the patient who declined chemotherapy, and who in addition was poorly compliant during radiotherapy despite repeated staff warnings with a two-week treatment break. Two of the three patients with local recurrence developed distant metastases following salvage TL. The third is still free of disease 30 months after treatment. The fourth just completes his surgery for salvage. All recurrences were detected on PET imaging, and proven by biopsy. In two patients with a subglottic recurrence and a second base of tongue primary, respectively, endoscopic examination was normal, with PET imaging leading to confirmation of recurrence. Another patient developed localized non-small cell lung cancer which was discovered on PET imaging and treated with stereotactic body radiotherapy. Three patients died at 11 to 14 months after treatment. The causes of death was stroke, second lung primary, and aspiration pneumonia, each respectively in one patient. The 2-year and 3-year survival is estimated to be 80% and 62.3% respectively for the whole group.

Twenty patients (74%) developed grade 3–4 toxicity during treatment, mainly mucositis (16 patients) and hematologic toxicity (4 patients). Two patient developed aspiration pneumonia, requiring brief intubation in one patient during the sixth week of radiotherapy. While all patients completed radiotherapy, fifteen (55%) had treatment breaks ranging from one to 29 days (median: 7 days) because of grade 3–4 toxicity. Median weight loss, despite prophylactic gastrostomy tube placement, was 9 pounds (0–25 pounds). Chemotherapy was not administered, or not administered according to the protocol in nine patients (33%). In addition to the patient declining chemotherapy, seven had schedule modifications due to side-effects during treatment. The patient receiving induction TPF developed bowel obstruction after two cycles, requiring discontinuation of the third cycle. The same patient subsequently developed pancytopenia (white blood cells: 1300, hemoglobin: 8 g/dl, platelets: 83,000) during chemoradiation requiring discontinuation of carboplatin after week 6 of radiotherapy.

Among patients for whom a standard cisplatin regimen of 100 mg/m2 on days 1, 22, and 43 was planned, one patient required a dose reduction to 80 mg/m2 for the first two cycles, and did not receive the third cycle because of aspiration pneumonia. Another patient also did not receive the third cycle because of neutropenia (white blood cells: 1,000). Another patient had cisplatin dose reduction to 75 mg/m2 after the second cycle because of renal dysfunction (Cr: 1.5, BUN: 36). Among patients receiving weekly cisplatin, two patients had protocol violations: cisplatin was delayed at week 6 in one patient because of neutropenia (white blood cells: 1,500) and discontinued after week 5 in another because of aspiration pneumonia. However, all patients completed the intended dose of radiotherapy despite treatment break.

At last follow-up, 22 of the 23 patients without recurrence reported normal or near normal voice quality, allowing them to communicate effectively at work or over the phone. The remaining patient reported persistent hoarseness and voice weakness, and had still been unable to work full time 12 months after treatment. His follow-up visits confirmed chronic laryngeal edema on endoscopic, with no sign of recurrence on PET imaging at 4 and 10 months after treatment. Among the entire group of 25 patients, four (16%) were still dependent on tube feedings at 6 to 16 months after treatment, due to severe dysphagia in 2 patients, and chronic aspiration in the other 2. Chemotherapy regimens in these four patients were weekly cisplatin in 2 patients, three-weekly cisplatin in one patient, and induction TPF followed by concurrent weekly carboplatin in one patient. The patient who received TPF/carboplatin finally died of aspiration pneumonia with no sign of recurrence 6 months after treatment. Table 4 summarizes acute and late toxicities following treatment for the whole group.

**Table 4:** Acute and late toxicities following intensity-modulated and image-guided radiotherapy for 27 patients with locally advanced laryngeal cancer.

Acute	Late
Grade 3–4 mucositis (16)	Long-term tube feedings (4)
Grade 3–4 hematologic toxicity (4)	Aspiration pneumonia (1)
Aspiration pneumonia (2)	
Bowel obstruction (1)	
Herpes Zoster (1)	

## Discussion

**Figure 2:**
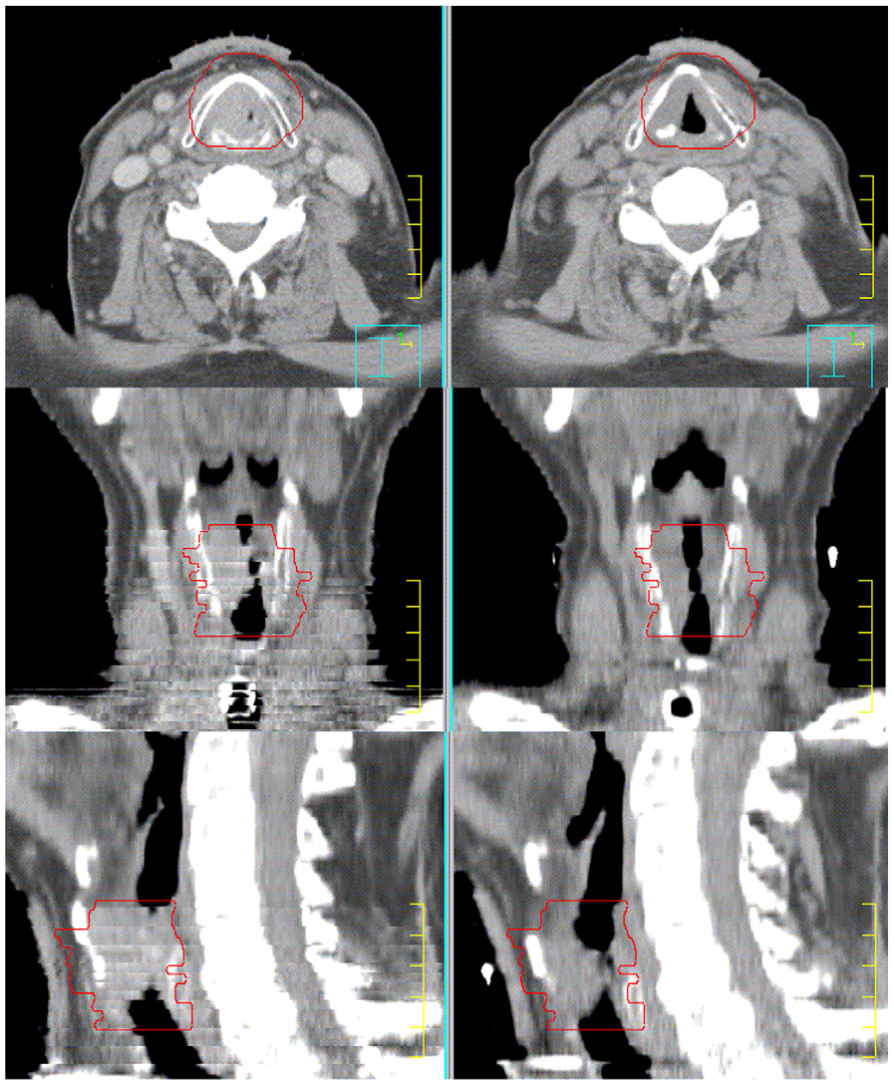
Illustrating the effectiveness of intensity-modulated and image-guided radiotherapy to achieve local control in T4 laryngeal cancer. The tumor invaded the thyroid cartilage and soft tissue of the neck and produced acute airway obstruction requiring emergency tracheostomy. A repeat CT scan at 40 Gy demonstrated significant shrinkage of the tumor allowing removal of the tracheostomy tube after treatment. The patient is disease-free 45 months after treatment and conserve a normal voice allowing him to work part-time after retirement.

To our knowledge, this is the first study looking at functional laryngeal preservation following chemoradiation for locally advanced laryngeal cancer with IMRT and IGRT. Even though our patient number was small and the follow-up relatively short, the anatomic laryngeal preservation rate we observed was similar to that reported in the RTOG 91-11 study, at 89% if we exclude the patient who declined chemotherapy and was non-compliant during radiotherapy. Most patients in the RTOG 91-11 had T2-T3 disease because it was thought that patients with T4 disease would have a non functional larynx following treatment [8]. Of note, six of the patients in our study had T4 disease and would thus have been excluded from concurrent chemoradiation in the RTOG study. All of these patients had no sign of recurrence at 11 to 45 months after treatment demonstrating the feasibility of organ preservation with IMRT and IGRT in T4 disease. For instance, one patient had disease invading the soft tissue of the neck and producing acute airway compression requiring emergency tracheostomy. The tumor shrank significantly during treatment allowing removal of the tracheostomy tube after treatment (Figure 2). The patient recovered his voice following treatment and, was able to resume his work without any difficulty with communication. PET imaging following treatment appeared in our patient group to be an effective follow-up strategy, detecting recurrence in two patients with normal endoscopic examination, thus confirming other studies reporting that PET imaging after radiotherapy for laryngeal cancer is helpful in detecting disease recurrence or second primaries, and thereby making laryngeal preservation therapy more feasible without compromising survival [23]–[25]. No patient with a negative PET scan in our study had disease recurrence, thus corroborating the accuracy of PET scan described in the literature [26]. In fact, in addition to the benefits provided by IMRT and IGRT, we feel that the excellent voice preservation observed in our patients, may in part be due to the avoidance of unnecessary biopsies in patients with negative PET imaging, reducing the edema and scarring frequently observed following laryngeal irradiation. In the recent past, laryngeal biopsy has been recommended in patients with edema persisting beyond six months following radiotherapy for laryngeal cancer [27]. However, recurrence is frequently submucosal and not visualized on direct laryngoscopic examinations and negative biopsy results requiring repeat procedures are common increasing the risk of chondritis and laryngeal necrosis [28], [29]. As reported in several studies, PET imaging has an excellent negative predicting value after treatment, and a negative PET exam in the presence of persistent laryngeal edema after radiotherapy is a strong argument that such edema is benign and not due to local recurrence. Because of the retrospective nature of the study, we did not generally have stroboscopy data available to assess vocal cord mobility. However, only one patient complains of hoarseness and weakness of his voice preventing him from having a full time job as he works as a salesman. He had chronic laryngeal edema which did not improve over time.

The rate of severe chronic dysphagia and aspiration we observed during and after treatment remains high and corroborated RTOG 91-11 data [8]. Among 172 patients randomized to concurrent chemoradiation, 77% of the patients developed severe grade 3–4 toxicity, 5% died from treatment toxicity, and 9% did not complete the intended dose of radiotherapy (70 Gy). At one year, 3% of these patients could not swallow at all, and 23% could only tolerate liquids or soft food. Our study acute grade 3–4 mucositis and late toxicity remained significant with concurrent chemoradiation despite the use of IMRT, an observation corroborated in other studies of IMRT in laryngeal cancers [30], [31]. Lee et al [30] reported a 35% rate of grade 3 mucositis and laryngitis in 31 patients with laryngeal and hypopharyngeal cancers undergoing concurrent chemoradiation with IMRT with six patients (19%) remaining dependent on long-term tube feedings. In another study of IMRT for laryngeal and hypopharyngeal cancer, eight out of 36 patients (25%) who had PEG tubes inserted before or during radiotherapy remained tube dependent more than 12 months after treatment. Given the close proximity of the pharyngeal musculature, severe dysphagia and aspiration remains unavoidable with the delivery of curative radiation doses to the larynx. Caglar et al demonstrated that the aspiration risk following IMRT for head and neck cancer was proportional to the volume of the inferior pharyngeal constrictor muscles receiving >50 Gy [32]. While it is thus not surprising that aspiration and long-term feeding-tube dependency remain prevalent after IMRT for laryngeal cancer, the aspiration risk is significantly lower following IMRT than following conventional radiotherapy with two lateral fields and a supraclavicular fields. Nguyen et al reported a 54% aspiration rate after concurrent chemoradiation for locally advanced laryngeal cancer with conventional radiotherapy [33]. In another study, 84% of the patients with definitive conventional radiotherapy for laryngeal cancer reported chronic aspiration [34]. Of note, aspiration after radiotherapy for head and neck cancer is often silent and can cause death from aspiration pneumonia if undetected [35], [36]. Compared to conventional radiotherapy, IMRT and IGRT provide more homogeneous dose coverage of target volume and steeper dose gradients, potentially decreasing the radiation dose to the pharyngeal muscles and thereby decreasing the aspiration rate [37]. Nonetherless, severe dysphagia and aspiration remain significantly high with IMRT and IGRT for locally advanced laryngeal cancer. Following treatment, swallowing therapy should be initiated early within four to six weeks if dysphagia or aspiration are present [38]. Another possible investigation is the use of Amifostine, a radiation protector, which has been proven to decrease late dysphagia following radiotherapy for head and neck cancer [39]. Amifostine should be considered in future prospective trials to investigate whether it can reduce aspiration rate [39].

The limitations of the present study include its retrospective nature, the small number of patients, the short follow-up, and the absence of a matched controlled group treated with 3-D conformal technique. However, it demonstrates the feasibility of modern radiotherapy techniques including IMRT and IGRT for anatomic and functional laryngeal preservation in patients with locally advanced laryngeal cancers. Further prospective studies with larger patient numbers should be performed with these techniques, in order to try confirm their positive impact on patient quality of life.

## Conclusions

New radiotherapy techniques such as IMRT and IGRT may be effective for anatomic and functional laryngeal preservation in patients with locally advanced laryngeal carcinoma. However, severe dysphagia and aspiration still remain limiting factors for surviving patients most likely secondary to excessive radiation dose to the pharyngeal muscles. Clinicians should be alerted about the risk of aspiration pneumonia for proper management.
